# Categorical principal component analysis to characterize patients at Intensive Care Unit admission

**DOI:** 10.1093/eurpub/ckac131.300

**Published:** 2022-10-25

**Authors:** G Favara, M Barchitta, A Maugeri, E Campisi, C La Mastra, MC La Rosa, R Magnano San Lio, I Mura, A Agodi, SPIN-UTI network

**Affiliations:** 1 Department G.F. Ingrassia, University of Catania, Catania, Italy; 2 Italian Study Group of Hospital Hygiene, Italian Society of Hygiene, Rome, Italy; 3 University of Sassari, Sassari, Italy

## Abstract

**Background:**

Healthcare-associated infections (HAIs) are the most frequent complications in healthcare settings, with a major impact on adverse outcomes. Here, we aimed to identify the relationships between patients’ characteristics admitted to Intensive Care Units (ICUs).

**Methods:**

We used data of patients included in the “Italian Nosocomial Infections Surveillance in Intensive Care Units” (SPIN-UTI) project, who stayed in ICU for more than 2 days. Using Categorical principal component analysis (CATPCA) two components of risk were assessed. Values of variance accounted for (VAF) >0.3 were accepted as the significant effect of a variable on each component. A Chronbach’s alpha >0.7 was accepted as a measure of the internal consistency of the model.

**Results:**

A total of 22402 admissions (62% female) were included. The average age was 65.7 years (SD = 16.6). Our model explains 35.3% of the total variability, with a Cronbach's alpha value of 0.847. The visual examination of component loading plot allows to evaluate the correlation between the quantified variables and each of the two components. In particular, the first component is explained by the presence of intubation (VAF=0.826), central venous catheter (VAF=0.749), and urinary catheter (VAF=0.727), patient’s origin (VAF=0.584), antibiotic treatment (VAF=0.479), non-surgical treatment for acute coronary disease (VAF=0.375), type of admission (VAF=0.509), surgical intervention (VAF=0.419). In the second component, the variables with the greatest contribution were the SAPS II (VAF=0.660), age (VAF=0.583), type of admission (VAF=0.531), surgical intervention (VAF=0.522). Thus, the first component would represent the exposure to invasive devices and medical procedures, and the second component the severity of patients.

**Conclusions:**

Our results proposed the usefulness of CATPCA to identify factors involved in the development of adverse outcomes, highlighting the role of exposure to invasive devices and severity of patients.

**Key messages:**

• There are several relationships between patients clinical and personal characteristics.

• CATPCA represents a useful approach for the analytical exploitation of healthcare data.

